# Gut microbiota dependant trimethylamine N-oxide and hypertension

**DOI:** 10.3389/fphys.2023.1075641

**Published:** 2023-04-06

**Authors:** Katongo H. Mutengo, Sepiso K. Masenga, Aggrey Mweemba, Wilbroad Mutale, Annet Kirabo

**Affiliations:** ^1^ HAND Research Group, School of Medicine and Health Sciences, Mulungushi University, Livingstone, Zambia; ^2^ Schools of Public Health and Medicine, University of Zambia, Lusaka, Zambia; ^3^ Department of Medicine, Levy Mwanawasa Medical University, Lusaka, Zambia; ^4^ School of Public Health, University of Zambia, Lusaka, Zambia; ^5^ Department of Medicine, Vanderbilt University Medical Center, Nashville, TN, United States

**Keywords:** hypertension, gut, microbiota, TMAO, trimethylamine N-oxide, mechanisms

## Abstract

The human gut microbiota environment is constantly changing and some specific changes influence the host’s metabolic, immune, and neuroendocrine functions. Emerging evidence of the gut microbiota’s role in the development of cardiovascular disease (CVD) including hypertension is remarkable. There is evidence showing that alterations in the gut microbiota and especially the gut-dependant metabolite trimethylamine N-oxide is associated with hypertension. However, there is a scarcity of literature addressing the role of trimethylamine N-oxide in hypertension pathogenesis. In this review, we discuss the impact of the gut microbiota and gut microbiota dependant trimethylamine N-oxide in the pathogenesis of hypertension. We present evidence from both human and animal studies and further discuss new insights relating to potential therapies for managing hypertension by altering the gut microbiota.

## Introduction

The gut microbiota is a community of micro-organisms consisting of approximately 100 trillion micro-organisms mainly bacteria, but also viruses, fungi, and protozoa that exist in the human gastrointestinal tract, interact with one another and with the host and greatly impact human health and physiology (Clemente et al., 2012). The gut microbiota are useful for the digestion of macronutrients and the production of a wide range of metabolites (Scott et al., 2013). Recent years have seen a growing interest in the gut microbiota and its potential impact on human health. One area of particular focus is the gut-associated metabolite trimethylamine N-oxide (TMAO) (Onyszkiewicz et al., 2020; Naqvi et al., 2021), which is produced when certain dietary components containing choline, l-carnitine, phosphatidylcholine and betaine are metabolized by the gut microbiota to yield trimethylamine (TMA). TMA is then rapidly oxidized to form TMAO in the liver by the enzymatic action of hepatic flavin-containing monooxygenase-3 (FMO3) (Koeth et al., 2013; Zeisel and Warrier, 2017).

Recently, high levels of TMAO have been found to be associated with an increased risk of cardiovascular disease (CVD) including heart failure, and chronic kidney disease (Zhang et al., 2021; Zhou et al., 2021). Emerging evidence suggests that TMAO may play a role in vascular endothelial dysfunction and hypertension (Seldin et al., 2016). The endothelium is a crucial regulator of cardiovascular health, with its proper functioning essential for maintaining optimal blood flow and blood pressure. Disruption of endothelial function has been linked to hypertension, a major risk factor for CVD. Current literature has shown that high levels of TMAO induces oxidative stress and inflammation in the vascular endothelium, which can impair endothelial nitric oxide (NO) production and bioavailability (Boini et al., 2017). NO is a potent vasodilator that regulates blood pressure by promoting relaxation of vascular smooth muscle cells. Impaired NO-mediated vasodilation can lead to endothelial dysfunction, vasoconstriction and elevated blood pressure (Cyr et al., 2020). TMAO has also been shown to promote endothelial cell apoptosis and disrupt endothelial barrier integrity, which can further contribute to vascular endothelial dysfunction and hypertension (Sun et al., 2016; Boini et al., 2017; Yang et al., 2019). Additionally, the activation of the NOD-like receptor protein 3 (NLRP3) inflammasome by TMAO has been suggested as one of the mechanisms for the development of endothelial dysfunction and hypertension (Chen et al., 2017). This occurs as TMAO triggers the production of pro-inflammatory cytokines and aggravates oxidative stress, leading to the osteogenic differentiation of vascular smooth muscle cells and subsequent vascular calcification, which contribute to the development of hypertension (Seldin et al., 2016; Boini et al., 2017; Chen et al., 2017; Wang et al., 2021).

Given the high prevalence of hypertension and its impact on cardiovascular health, a deeper understanding of the mechanisms involved in the aetiology and potential therapeutic targets is crucial. By exploring the complex relationship between TMAO, gut microbiota, and hypertension, we can gain new insights into the pathogenesis of hypertension and develop novel strategies to prevent or treat this condition. In this review, we provide insights into the current understanding of the host-gut microbiota interaction *via* the TMAO metabolite and summarize evidence of its importance in the pathogenesis of CVD and hypertension.

## Composition and function of the human gut microbiota

Although several bacterial species occupy the gut, most evidence point to the fact that total composition of the healthy gut microbiota largely comprises species from the Bacteroidetes (predominantly gram negative *Bacteroides* or Prevotella) and the Firmicutes (predominantly gram positive *Clostridium* and *Lactobacillus*) phyla, respectively (Qin et al., 2010; Rinninella et al., 2019a; King et al., 2019), and to a lesser extent Actinobacteria and Proteobacteria (Rinninella et al., 2019b). However, there may be some variance in the ratios of these gut microbiota. For example, a study in mice showed that the abundance of Firmicutes decreases from the stomach to faeces, while Bacteroidetes gradually increased with higher diversity in the colon and cecum (Lkhagva et al., 2021). Similarly, human samples from the small intestines were shown to be enriched in the *Bacilli* class of the Firmicutes and Actinobacteria, while the Bacteroidetes and the Lachnospiraceae family of Firmicutes were more abundant in colonic samples (Frank et al., 2007; Sekirov et al., 2010). The earlier problem of culturing gut microbiota to identify the species has now been overcome by the latest molecular techniques such as metagenomics, which produces a taxonomical profile of the sample and metabolomics, the large-scale study of metabolites (by-products of metabolism) within cells, biofluids, tissues or organisms (Aguiar-Pulido et al., 2016).

A symbiotic relationship exists between the gut microbiota and the gut mucosa which imparts substantial metabolic, immunological and gut protective functions in the healthy individual (Jandhyala et al., 2015). The gut microbiota provides various functions, including aiding in digestion, regulating the immune system, and producing vitamins such as vitamin D and other B vitamins, as well as other essential nutrients (Morowitz et al., 2011). The nutrients are critical for various bodily functions, such as blood clotting, bone health, and neural systems’s functioning. Bacteria phyla such as Firmicutes break down complex carbohydrates which are not easily digested the human body into short-chain fatty acids, promoting nutrient absorption while Actinobacteria and Proteobacteria are known to be involved in production of vitamin B12 (Rowland et al., 2018). On the other hand, Bacteroidetes break down complex polysaccharides and play a role in vitamin production and regulating inflammation in the gut (Belkaid and Hand, 2014; Rowland et al., 2018). Furthermore, the gut microbiota is involved in the synthesis of neurotransmitters, such as serotonin and dopamine, resulting in mood regulation and other physiological functions (Chen et al., 2021). Some gut microbiota associated metabolites have antidiabetic, antiatherogenic, obesity and lipid lowering and immunomodulatory properties (Jandhyala et al., 2015; Chia et al., 2020; Nogal et al., 2021). Additionally, gut microbiota have also been shown to be involved in the metabolism of drugs which might affect their therapeutic function (Yoo et al., 2016) including antihypertensive drugs (Mishima and Abe, 2022). The gut microbiota also increases villous vascularization, leading to improved nutrient absorption (Bäckhed et al., 2004), and therefore is important in the overall wellbeing. In exchange, the human body provides microbiota with a stable environment and a constant supply of nutrients for the microorganisms to thrive (Mondot et al., 2013).

Human and animal studies suggest that several bacteria taxa are involved in trimethylamine and TMAO production, but predominantly those from the Firmicutes phylum (Romano et al., 2015; Rath et al., 2017; Beale et al., 2019). Various bacterial taxa belonging mostly to the Firmicutes phylum such as Clostridia, Bacilli, Desulfovibrionia, and Proteobacteria such as Gammaproteobacteria contain the choline trimethylamine-lyase (*CutC*) gene and are significant producers of trimethylamine, a precursor of TMAO (Romano et al., 2015; Rath et al., 2017), while bacteria belonging to Bacteroidetes phylum are not able to produce trimethylamine (Janeiro et al., 2018). As mentioned earlier, the gut microbiota predominantly comprise the Firmicutes and Bacteroidetes (Qin et al., 2010; Rinninella et al., 2019a; King et al., 2019) and a high Firmicutes to *Bacteroides* ratio is associated with gut dysbiosis (Beale et al., 2019).

## TMAO physiological and pathophysiological actions

The biosynthesis and metabolism of TMAO has been previously described in detail (Liu and Dai, 2020). Briefly; TMAO is biosynthesized from trimethylamine, which is produced as result of the action of the gut microbiota on nutritional substrates containing phosphatidyl-choline (lecithin), choline, L-carnitine and betaine (Koeth et al., 2013; Velasquez et al., 2016). Dietary derivatives of these compounds include red meat, liver, fish, dairy products, and eggs, whereas sources of betaine (a choline oxidation product) also include wheat bran, wheat germ, and spinach (Senthong et al., 2016). Intestinal bacterial strains with trimethylamine lyases convert these nutritional substrates to trimethylamine (Maffei et al., 2022). Trimethylamine from the gut reaches the liver by portal circulation, where host hepatic FMO3 incorporates oxygen atoms into trimethylamine to form TMAO (Gątarek and Kałużna-Czaplińska, 2021). TMAO is then either transported to the tissues for accumulation as an osmolyte, or excreted through the common route *via* the kidney (Meyer et al., 2016; Gątarek and Kałużna-Czaplińska, 2021). Other ways of excretion of TMAO are through sweat, faeces (4%), exhaled air (less than 1%) or other body secretions (Papandreou et al., 2020). TMAO can also be degraded to methylamine, dimethylamine, methane, and ammonia within the colon (Chhibber-Goel et al., 2016; Velasquez et al., 2016). Dietary choline can be incorporated into larger molecules such as the lipid phosphatidyl-choline, but choline-utilizing gut microbiota can hydrolyse phosphatidyl-choline using a phospholipase D (PLD) enzyme and further convert the released choline to trimethylamine (Chittim et al., 2019).

TMAO was first identified in marine crustaceans and marine fish and has been suggested to play a role in reducing osmoregulatory costs, increase buoyancy, or counteract destabilization of proteins by pressure (Kelly and Yancey, 1999). Deep-sea animals have been shown to accumulate TMAO under physiological conditions to protect enzymatic activity such as lactate dehydrogenase (LDH) from hydrostatic and/or osmotic pressure stress (Gawrys-Kopczynska et al., 2020). TMAO is also observed to have a protective effect in animals (Huc et al., 2018). Huc et al. (2018) showed that chronic, low-dose TMAO that increases plasma TMAO by four- to five-fold, reduced left ventricular end-diastolic pressure, and cardiac fibrosis in pressure-overloaded hearts in hypertensive rats, and that higher levels of TMAO had no negative effects on circulation. However, human studies have reported mixed results (Tang et al., 2013; Meyer et al., 2016), although increasing evidence points to a propensity towards risk of adverse cardiac events and cardiovascular risk factors such as hypertension associated with elevated plasma TMAO levels (Tang et al., 2013; Senthong et al., 2016; Smiljanec and Lennon, 2019; Wang et al., 2019). TMAO also appears to participate in the pathologic processes of heart failure (HF) and can serve as an early warning marker to identify individuals who are at the risk of disease progression (Zhang et al., 2021). Preclinical experiments show that TMAO may directly affect the heart by inducing cardiac mitochondrial dysfunction, endothelial cell and vascular inflammation, and finally myocardial hypertrophy and fibrosis (Sun et al., 2016; Makrecka-Kuka et al., 2017; Li et al., 2019). Processes like vascular inflammation and myocardial hypertrophy/fibrosis may lead to hypertension and cardiac events like heart failure respectively (de Leuw et al., 2021; Pugh and Dhaun, 2021).

## TMAO and hypertension mechanisms

The mechanisms by which TMAO increases gut microbiota-mediated CVD risks are multifactorial, although inflammation seems to be central to this association (Liu and Dai, 2020; Huang et al., 2022). The link between TMAO and CVD was first made using an animal model in 2011 by Wang et al. (2011), which revealed that choline, TMAO and betaine were predictors of CVD risk. Wang et al. (2022) have recently demonstrated a causal relationship of TMAO and its precursors with blood pressure using a Mendelian Randomization approach. Several studies have now demonstrated a close relationship between high TMAO levels and a higher prevalence of hypertension (Senthong et al., 2016; Suzuki et al., 2016; Ge et al., 2020). Ge et al. (2020) reported that an increase of 5 and 10 μmol/L in TMAO level was associated with a 9% and 20% increase in the risk of hypertension, respectively.

Several potential mechanisms by which TMAO promotes hypertension (discussed in detail in order below the list) include:

• enhance Ang II-induced vasoconstriction and acute pressor response *via* activating the PERK pathway leading to apoptosis, inflammation, and vascular injury resulting in hypertension (Montezano and Touyz, 2012; Sinha and Dabla, 2015; Jiang et al., 2021)
• upregulating of scavenger receptors on the surface of macrophages and promoting foam cell formation, atherosclerosis, vascular constriction and arterial stiffness (Canyelles et al., 2018; Liu and Dai, 2020; Brunt et al., 2021; Jiang et al., 2021)
• interfering with the reverse transport of cholesterol from extrahepatic organs and tissues into the liver resulting in increased oxidised-low density lipoprotein (ox-LDL) deposition in peripheral tissues (Canyelles et al., 2018; Wang et al., 2021), a process that accelerates atherosclerosis and CVD.
• Increasing IL-1β, IL-18, and tumor necrosis factor-α, while decreasing the expression of anti-inflammatory cytokines such as interleukin-10 (Liu and Dai, 2020). TMAO also significantly increased the expression of proinflammatory cytokines and chemokines including *Kng1*, *Cxcl1*, *Cxcl2*, *Cxcl6*, and *Il6* in bone-marrow-derived macrophage (Huang et al., 2022).
• Indirectly by promoting renal dysfunction (Zhou et al., 2021)
• Promoting cardiovascular dysfunction at higher plasma levels (Naghipour et al., 2021) through induction of cardiac mitochondrial dysfunction, myocardial hypertrophy and fibrosis (Sun et al., 2016; Makrecka-Kuka et al., 2017; Li et al., 2019).
• Salt induced TMAO production resulting in hypertension

### PERK pathway activation

In a landmark study by Chen et al. (2019), they found that TMAO directly bound to and activated protein kinase R-like endoplasmic reticulum kinase (PERK); the endoplasmic reticulum (ER) stress kinase which has been associated with vascular injury *via* apoptotic inflammatory responses and ROS generation leading to increased risk for hypertension (Montezano and Touyz, 2012; Sinha and Dabla, 2015). ER stress occurs when there are physiological stressors which lead to failure of ER functions, specifically a decrease in the protein-folding capacity and subsequent accumulation of undesirable unfolded or misfolded proteins in the ER lumen (Shen et al., 2004). However, the ER has evolved stress response signalling pathways to maintain ER homeostasis collectively known as the unfolded protein response (UPR) (Shen et al., 2004).

UPR involves signal transduction pathways which reprogram gene transcription, mRNA translation and protein modifications to relieve the load of unfolded or misfolded proteins and restore protein homeostasis (proteostasis) (Hetz et al., 2020). In the event of chronic damage or chronic stress, the UPR induce apoptotic cell death (Shen et al., 2004; Chen et al., 2019; Hetz et al., 2020). PERK is one of the three UPR signaling pathways, with other two being the inositol-requiring protein 1α (IRE1α) and activating transcription factor 6α3 (ATF6α3) (Ron and Walter, 2007; Chen et al., 2019). PERK kinase phosphorylates eukaryotic translation initiation factor 2 subunit-α (eIF2α) (Ron and Walter, 2007; Hetz et al., 2020). This phosphorylated eIF2α enables the translation of *Atf4* mRNA, a transcription factor which promotes adaptation to stress and transiently halt protein synthesis and release in the ER, but can also lead to the activation of apoptosis during prolonged, unresolved stress (Ron and Walter, 2007; Lin et al., 2009; Chen et al., 2019). UPR is therefore a double-edged sword as an adaptive response and also a maladaptive response associated chronic activation. Sustained PERK activation has been implicated in impaired cell proliferation and promotion of apoptosis (Lin et al., 2009). Apoptosis promotes inflammatory responses, nicotinamide adenine dinucleotide phosphate (NADPH) oxidase activation, and ROS generation which all lead to vascular damage, and sets a precedence to hypertension (Montezano and Touyz, 2012; Sinha and Dabla, 2015), Figure 1.

**FIGURE 1:**
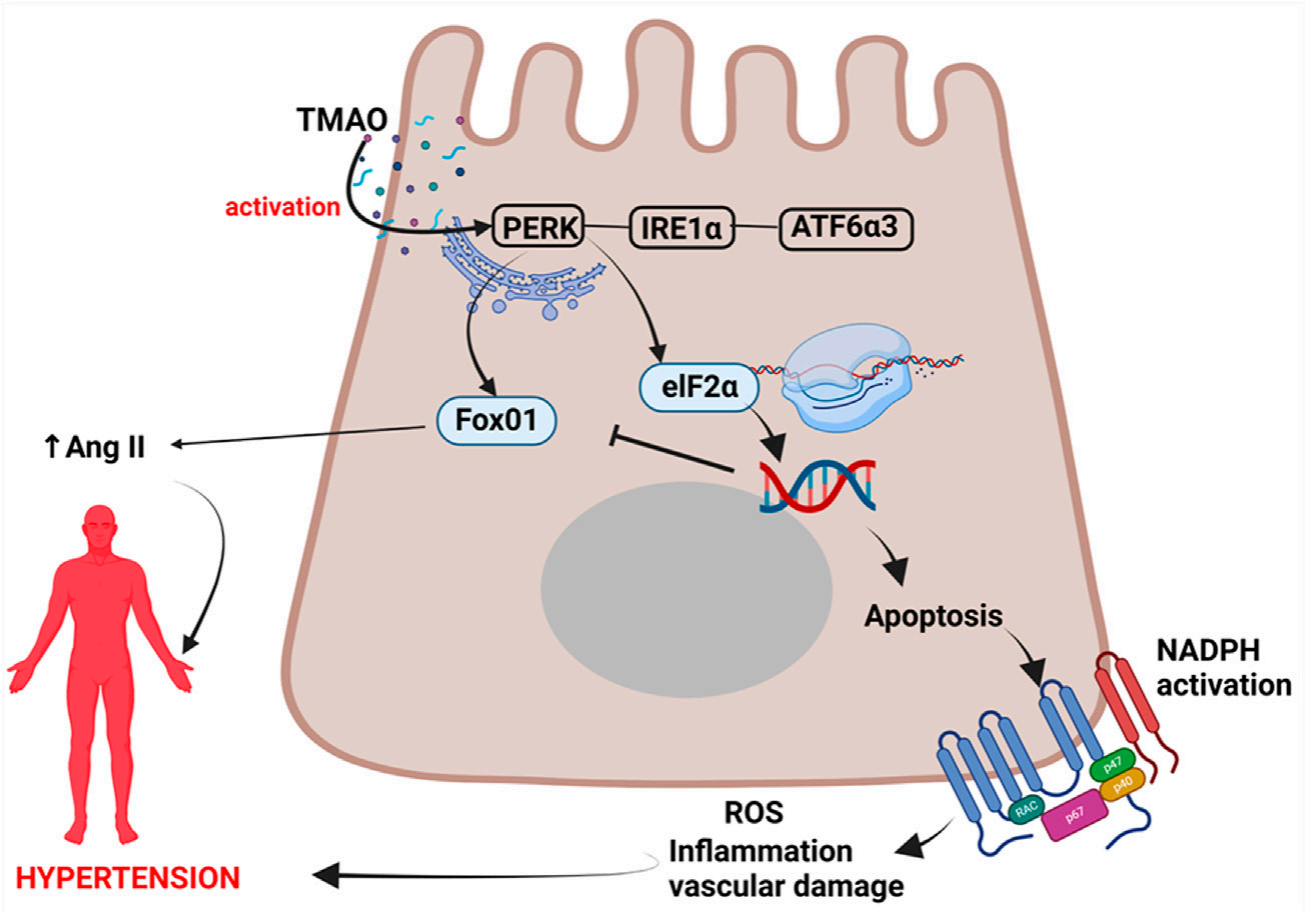
Mechanism of TMAO activation of PERK pathway leading to hypertension. TMAO activates the PERK pathway leading to apoptosis, inflammation, and vascular injury resulting in hypertension. TMAO, trimethylamine N-oxide; IRE1α, inositol-requiring protein 1α; ATF6α3, activating transcription factor 6α3; eIF2α, eukaryotic translation initiation factor 2 subunit-α; NADPH, nicotinamide adenine dinucleotide phosphate oxidase; ROS, reactive oxygen species; Ang II, angiotensin II.

TMAO selectively activates the PERK pathway of the UPR and the PERK in turn induces FoxO1. This response is attenuated by terminating FMO3 function, an enzyme that activates TMAO, as well as by manipulating gut microbiota that may have enzymatic pathways leading to TMAO production (Chen et al., 2019). This indicates that gut microbiota indirectly induces FoxO1. FoxO1 belong to a group of transcription factors of the O class (FOXOs) having four members (Kousteni, 2010). FoxO1 is the main target of insulin signaling and regulates metabolic homeostasis in response to oxidative stress (Kousteni, 2010). While some evidence from animal studies have found FoxO1 to be protective to the heart (Yu et al., 2020), others have reported that increasing FoxO1 expression increase angiotensinogen and angiotensin II levels, which are important in the pathogenesis of hypertension (Qi et al., 2014). It is possible that chronic elevation of TMAO has the potential to chronically activate the PERK pathway and FoxO1 expression, and possibly contribute to the development of hypertension.

### Scavenger receptor activation and atherosclerosis

TMAO can contribute to hypertension *via* promotion of atherosclerotic processes such as formation of foam cells by upregulation of macrophage scavenger receptors (Wang et al., 2011). TMAO is increased in persons with advancing atherosclerosis compared to healthy persons (Tang et al., 2013). Experimental studies in mice suggest a causal association between TMAO plasma levels and atherosclerosis (Wang et al., 2011) and have demonstrated that TMAO induces expression of CD36 and scavenger receptor A1 (SR-A1) in activated macrophages thus stimulating the uptake of ox-LDL and foam cell formation (Yang et al., 2019; Liu and Dai, 2020). SR-A1 has been implicated in vascular injury and atherosclerosis by mediating endocytosis of foam cells on low-density lipoprotein (Watanabe et al., 2013), a process that accelerates atherosclerosis. Increased expression of SR-A1 on macrophages is dependent on nuclear factor kappa-light-chain-enhancer of activated B cells (NF-κB) pathway. Activation of NF-κB pathway is responsible for macrophage 1 polarization [classically activated macrophage (M1)] which secrets inflammatory cytokines such as IL-1, IL-6, IL-12, Tumour necrosis factor alpha (TNF-α), chemokines such as Monocyte chemoattractant protein-1 (MCP-1), IL-18, Regulated upon Activation, Normal T cell Expressed, and Secreted (RANTES), Macrophage inflammatory protein-2 (MIP-2), C-X-C Motif Chemokine Ligand 1 (CXCL1) and C-X-C motif chemokine ligand 10 (CXCL10) and adhesion molecules such as Intercellular Adhesion Molecule 1 (ICAM-1) and Vascular cell adhesion protein 1 (VCAM-1) (Liu et al., 2017). M1 macrophages promote differentiation of inflammatory T cells, including Th1 and Th17 cells, which in turn mediate inflammation (Wang et al., 2014). These processes exacerbate atherosclerotic plaques leading to vascular injury and constriction and infiltration of Th17 cells in the kidney with production of IL-17A activates the renin-angiotensin aldosterone system (RAAS) resulting in high blood pressure (Yang et al., 2022a; Ertuglu and Kirabo, 2022). Moreover, IL-17A in mice models has been reported to increase monocyte adhesion to the aortic wall and enhance endothelial cell pro-inflammatory cytokine production (Nordlohne et al., 2018). In this way, TMAO contributes to inflammatory cytokine production and pathogenesis of atherosclerosis and hypertension (Yang et al., 2019; González-Correa et al., 2021).

### Reverse cholesterol transport interference

Increased TMAO is known to decrease reverse cholesterol transport (RCT) resulting in increased deposition of cholesterol in arterial blood vessel and activation of scavenger receptors and atherosclerotic processes (Yang et al., 2019). TMAO represses bile acid synthesis in the liver, the major pathway for cholesterol elimination, by inhibiting Cyp7a1 expression in the classical pathway of bile acid synthesis accelerating the formation of aortic atherosclerosis (Zárate et al., 2016; Ding et al., 2018). In the liver, FMO3 that generates TMAO is linked to the processes resulting in reduced RCT as well (Yang et al., 2019). However, the mechanisms underlying RCT are still elusive. A detailed review of the relationship between high density lipoprotein (HDL) responsible for RCT and TMAO has been elaborated in detail elsewhere (Canyelles et al., 2018).

### Pro- and anti-inflammatory cytokine production

As discussed above, TMAO induces activation of the NF-kB resulting in the generation of proinflammatory cytokines that contribute to atherogenesis and vascular injury and remodelling (Seldin et al., 2016). TMAO also upregulates the nod-like receptor family pyrin domain containing 3 (NLRP3) inflammasome formation (Chen et al., 2017), and increases serum levels of the proinflammatory lipopolysaccharide (LPS) endotoxin (Maffei et al., 2022). NLRP3 Inflammasome leads to increased production of inflammatory cytokines and this has been demonstrated in carotid artery endothelial cells (CAECs), human aortic endothelial cells (HAECs) and vascular smooth muscle cells (VSMCs) suggesting that NLRP3 initiates an endothelial inflammatory response cascade leading to endothelial dysfunction (Seldin et al., 2016; Boini et al., 2017). Several studies have demonstrated that plasma TMAO correlates positively with plasma inflammatory cytokines. This was demonstrated in obese mice induced by feeding a western diet where TMAO levels increased along with TNF-α and IL-1β, with decrease in anti-inflammatory cytokine IL-10 (Chen et al., 2017). In human studies, increased plasma TMAO levels correlated with increased plasma levels of TNF-α, sTNF-R p75, and sTNF-R p55 in 271 German adults (Rohrmann et al., 2016) and IL-1β and hsCRP levels in 81 patients with stable angina (Chou et al., 2019).

### Indirect renal dysfunction

TMAO has been implicated in contributing to development of renal insufficiency and mortality risk in chronic kidney disease patients by directly inducing progressive renal fibrosis and dysfunction (Wilson Tang et al., 2015). TMAO is easily filtered by the kidney owing to its low molecular weight and is a nitrogenous waste product. Its plasma levels increase correlating with creatinine and urea levels (Pelletier et al., 2019). Increase in TMAO has been demonstrated to aggravate Angiotensin II-induced hypertension by inducing constriction of afferent arterioles and mesenteric arteries and mediating acute pressor response through the PERK/ROS/CaMKII/PLCβ3/Ca2+ signalling pathway in mouse (Jiang et al., 2021). This promotes oxidative stress through production of ROS contributing to kidney dysfunction and tubular damage (Jiang et al., 2021). TMAO-induced ROS production enhances the activity of calmodulin-dependent protein kinase II (CaMKII) which enhances Ang II response causing increased blood pressure. This is further aggravated by the production of IL-17A by activated Th17 cells, activation of NLRP3 and other mechanisms as earlier explained above (Kapetanaki et al., 2021). The mechanistic role of salt on kidney injury is elaborated in detail later.

### Cardiovascular dysfunction

TMAO induces aortic stiffening and increases systolic blood pressure *via* formation of advanced glycation end-products (AGEs) and superoxide-stimulated oxidative stress, which in consent increases intrinsic wall stiffness and can occur with the aging process (Brunt et al., 2021). This experimental human and mice study by Brunt et al. (2021) demonstrated that TMAO directly increases AGEs which then cross-link with structural proteins to mediate TMAO-induced aortic wall stiffness. Vascular injury from AGEs and oxidant stress results in and enhances platelet function. TMAO induces platelet activation *via* the second messenger Inositol trisphosphate causing hyperactivity and aggregation resulting in activation of hypercoagulable state (Zhu et al., 2020). This in turn results in thrombotic events that can contribute to stroke, heart attack or heart failure. TMAO also enhances platelet function in a dose dependant manner such that platelet reactivity is rapid and reversible (Zhu et al., 2020). Activation and hyperactivity of platelets occurs through interactions with vascular receptors or by soluble platelet agonists such as von Willebrand factor, collagen, and adenosine diphosphate (ADP), thrombin, and thromboxane A2, respectively (Li et al., 2010).

In large cohort of older US adults, TMAO was associated with higher risk of incident atherosclerotic cardiovascular disease (ASCVD) (Lee et al., 2021). In other studies, increased plasma TMAO was associated with increased risk of cardiovascular death, poor peripheral artery disease (PAD) prognosis (Roncal et al., 2019), heart failure (HF) *via* the gut–TMAO–HF axis (Zhang et al., 2021) and functionally relevant coronary artery disease (Amrein et al., 2022). However, the exact mechanisms of CVD development induced by TMAO are still unclear.

### Dietary salt and trimethylamine N-oxide in salt-sensitive hypertension

Hypertension can also be driven by high dietary salt (sodium) intake, hence the term salt-sensitive hypertension (Mishra et al., 2018). Salt sensitivity is an increase in blood pressure in response to high dietary salt intake (Pilic et al., 2016). High dietary sodium consumption may contribute to gut dysbiosis by altering the gut microbial composition, and inducing an inflammatory response which alters the gut anatomy and function (Smiljanec and Lennon, 2019; Elijovich et al., 2020), and is associated with increased immunogenic Isolevuglandins (IsoLG)-adducted protein formation responsible for upregulation of epithelial sodium channels and salt sensitivity (Ferguson et al., 2019; Van Beusecum et al., 2019). Sodium increases intracellular reactive oxygen species (ROS) by activating reduced NADPH oxidase resulting in arachidonic acid peroxidation which further leads to formation of IsoLG-adducted proteins (Xiao et al., 2018). IsoLG-adducted proteins may be presented by dendritic cells (DCs) as neoantigens that activate T-cells to infiltrate the kidney and vasculature; releasing cytokines that promote vascular dysfunction, including sodium and water retention (Xiao et al., 2018; Ferguson et al., 2019), processes that are key in the pathogenesis of hypertension.

TMAO has been demonstrated to be affected by high dietary salt intake (Bielinska et al., 2018). A study in Sprague–Dawley rats showed that high dietary salt caused a significant elevation in plasma TMAO and significantly lower 24-h TMAO urine excretion in mice on saline solutions than the controls on water (Bielinska et al., 2018). The female sex hormone; estradiol may act in two ways to regulate blood pressure. Firstly, estradiol interacts with the G protein–coupled receptor-1 (GPER1) promoting aldosterone secretion, and secondly the estrogen receptor-β (ERβ) reducing aldosterone secretion respectively (Caroccia et al., 2014). Experimental studies have showed that the blockade of ERβ result in unmasking a potent GPER-1-mediated secretagogue effect of estradiol (Caroccia et al., 2014), suggesting that some premenopausal women may have a predisposition to salt sensitivity due to aldosterone effects. Additionally, there are polymorphic variants of the ERβ that are associated with increased salt-sensitive BP due to inappropriate aldosterone levels on a salt diet (Manosroi et al., 2017).

As earlier mentioned, gut dysbiosis and reduction in α-diversity of gut microbiota reduce β-glucuronidase activity and result in decreased deconjugation of estrogen and phytoestrogen in circulation and active forms (Baker et al., 2017; Qi et al., 2021). Additionally, the gut microbiota has been shown to be altered with a skew toward dysbiosis on high dietary salt (Smiljanec and Lennon, 2019). A human and mice study by Ferguson et al found that a high-salt diet was associated with changes in the gut microbiome reflecting an increase in Firmicutes colonization with a resultant increase in Firmicutes/Bacteroidetes ratio (Ferguson et al., 2019). This was also accompanied by a synchronized increase in vascular inflammation and hypertension (Ferguson et al., 2019). The Firmicutes phylum (especially the *Clostridium* and Bacilli species) are trimethylamine producing gut bacteria, a precursor to TMAO (Jose and Raj, 2015; Romano et al., 2015; Rath et al., 2017). Furthermore, high dietary salt has also been shown to alter the profile of gut microbiota profile reflecting the low resilience of *Lactobacillus* species (Wilck et al., 2017). An animal study showed that depletion of the specific species *Lactobacillus murinus*, found only in mice, was significant when mice were fed a high dietary salt and reversed when they returned to the normal diet (Wilck et al., 2017). This depletion was associated with activation of T helper 17 lymphocytes (T_H_-17), the major modulators of the immune response leading to a proinflammatory state (Wilck et al., 2017). IL-17 and oxidative stress produced by T_H_17 cells lead to vascular injury and salt-sensitive hypertension (Toral et al., 2019). Activation of T_H_17 cells and hypertension in salt-sensitive mice fed with high dietary salt was curbed by supplementing the gut with *L. murinus* or *Lactobacillus reuteri*, suggesting the influence of these bacteria on blood pressure regulation (Wilck et al., 2017). In humans, a high salt diet reduced intestinal survival of *Lactobacillus species*, increased T_H_17 cells and increased blood pressure (Wilck et al., 2017). However, the *Lactobacillus* species are not included among those containing the choline trimethylamine-lyase (*CutC*) and its activating enzyme *CutD* gene responsible for TMAO production (Kalnins et al., 2015; Romano et al., 2015; Rath et al., 2017).

**FIGURE 2:**
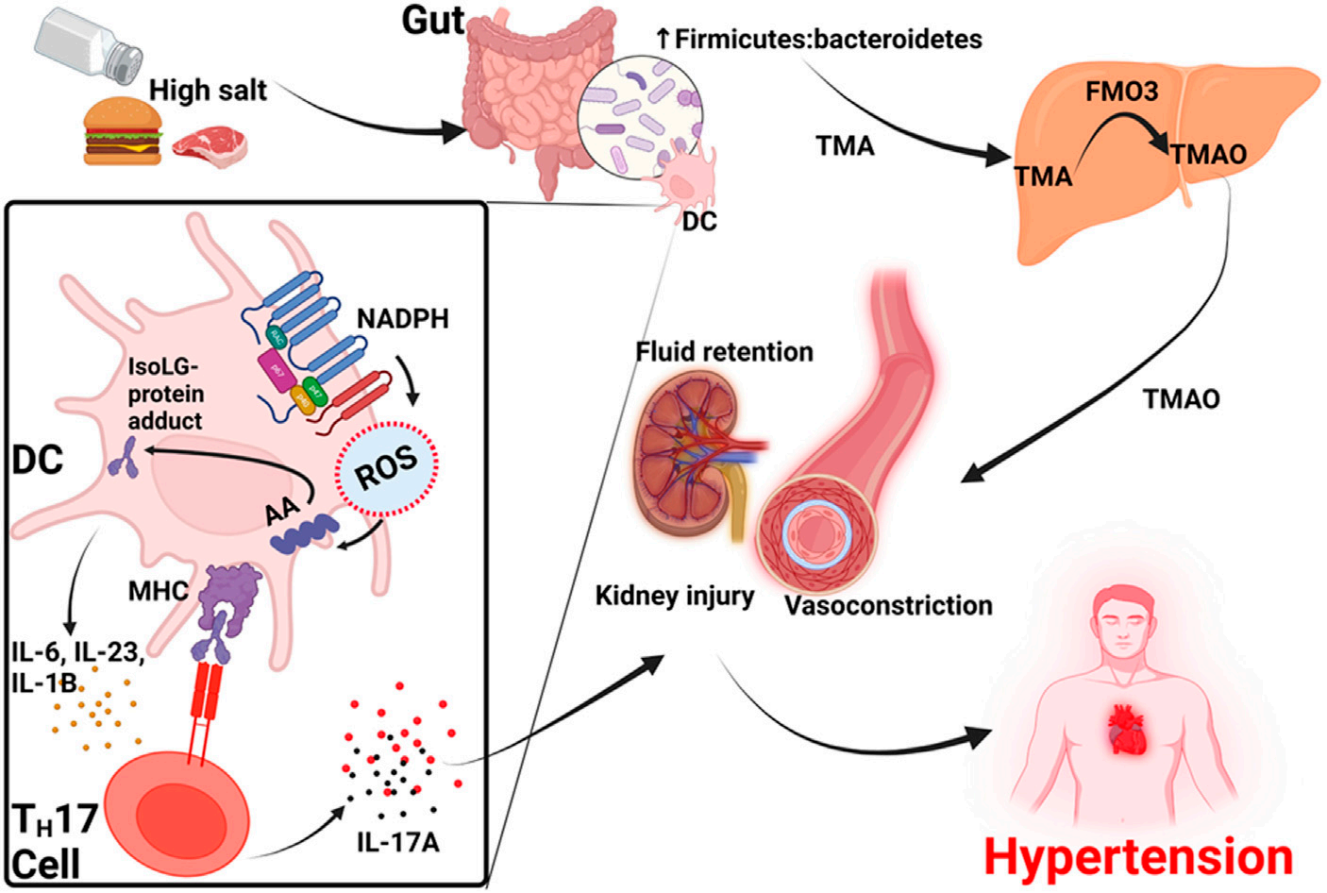
Proposed model for TMAO-induced hypertension in high salt diet. TMA, trimethylamine; DC, dendritic cell, TMAO, trimethylamine N-oxide; FMO3, flavin-containing monooxygenase-3; IsoLG, Isolevuglandin; AA, arachidonic acid; MHC, major histocompatibility complex; ROS, reactive oxygen species; T_H_17 cell, T-helper 17 cell; IL1B, Interleukin-1 beta; IL17A, Interleukin-17A.

These data suggests that high salt diets can increase blood pressure by three mechanisms. The first is by increasing gut Firmicutes: *Bacteroides* ratio resulting in increased TMA production in the gut that is later converted to TMAO in the liver. TMAO then contributes to constriction of afferent arterioles and mesenteric arteries and increase blood pressure (Jiang et al., 2021). The second mechanism is through activation of dendritic cells which activates NADPH oxidase to produce ROS that contribute to the formation of IsoLG-protein adducts that activate T cells to produce IL-17 and other cytokines. Activated T cells infiltrate the kidney and IL-17 promotes vascular injury and hypertension. Thirdly, dietary salt directly causes fluid retention in the kidney and high blood pressure, Figure 2.

## Nutrition and dietary factors modulating gut microbiota

The gut microbiota content can be influenced by dietary choices, as red meat is one of the major sources of TMAO production (Cho et al., 2017). In a German adult population, plasma concentrations of TMAO was directly associated with dairy food consumption such as milk and Low-Grade inflammation (Rohrmann et al., 2016). It must be highlighted that reduced levels of TMAO have been observed in vegetarians and vegans due to low amounts of L-carnitine and choline consumed along with food (Koeth et al., 2013). These dietary habits are not only key drivers in TMAO production (Wang et al., 2019), but gut dysbiosis as well (Valdes et al., 2018). Gut dysbiosis is linked to the development of CVD risks such as atherosclerosis and hypertension (Jose and Raj, 2015; Adnan et al., 2017; Avery et al., 2021; Canale et al., 2021).

Diet is able to modulate the gut microbiota structure by more than 50% (Zhang et al., 2010). The main conduit for gut-associated CVDs is mainly through inflammation elicited by dysbiosis from particular diets. For example, diets that mainly consist of fats and protein such as red meat and reduced intake of vegetables and high salt typical of the western diet is associated with gut dysbiosis resulting in increased risk for arterial hypertension (Canale et al., 2021). The underlying mechanisms include activation of various pathways that lead to vasoconstriction, sodium and water retention and continuous suboptimal inflammation resulting in hypertension (Canale et al., 2021). Among the mechanisms that contribute to dysbiosis-mediated high blood pressure include stimulation of hormonal secretion such as dopamine, serotonin and norepinephrine (Kang and Cai, 2018), and activation of the SCFA olfactory receptor 78 (Olfr78) expressed in the glomerular afferent arterioles and smooth muscle cells of arterial walls eliciting vasoconstriction activities partly through renin production resulting in increased blood pressure (Tang et al., 2017; Canale et al., 2021).

One of the most common diets associated with reducing the risk for CVDs is the Mediterranean diet that consist (as foundation of the diet) mainly of plant-based foods, such as whole grains, vegetables, fruits, seeds, legumes, nuts, herbs, spices and extra virgin olive oil (Trichopoulou et al., 2014; Muscogiuri et al., 2022). The consumption of fish, poultry, red meat, and dairy products is limited in the Mediterranean diet (Muscogiuri et al., 2022). This diet is associated with an increase in the production of SCFAs due to high fibre content and has been associated with reduction in cardiometabolic conditions including hypertension (Donohoe et al., 2012; Haro et al., 2016). A recent clinical trial by Xue et al. (2021) demonstrated that increased supplementation with dietary fibre from oat bran improved 24 h ambulatory blood pressure, reduced the amount of antihypertensive drugs required to lower blood pressure and modulated gut microbiota by increasing the relative abundance of *Bifidobacterium* and *Spirillum*. Based on several human and animal studies, there is evidence that long term dietary supplements can modulate the gut microbiota to ameliorate hypertension. Examples include dietary fibre (Marques et al., 2017; Snelson et al., 2021; Xue et al., 2021), extra virgin oil (Hidalgo et al., 2018), some herbs (Panyod et al., 2021) and garlic oil among others (Hsu et al., 2021).

Although the current relationship between the Mediterranean diet and specific gut microbiota associated with hypertension from literature is not yet established to be a cause-to-effect, this diet is reported to increase biodiversity or what is termed as alpha diversity of the gut microbiota associated with reduced CVD risk (Merra et al., 2020). Reduced alpha diversity is generally associated with poor health outcomes including hypertension (Walter and Ley, 2011; Verhaar et al., 2020). Research by the METAgenomics of the Human Intestinal Tract (MetaHIT) showed that richness and diversity of gut microbiota genes correlates with metabolic markers (Le Chatelier et al., 2013). In this study, they found that gene quantity, and hence bacterial richness stratified as “low gene count” and “high gene count” was influenced by diet and determined overall metabolic abnormalities such as weight gain, insulin resistance, and dyslipidemia, including a proinflammatory state. Individuals with low gene count had a propensity towards abnormal metabolic parameters and proinflammatory state compared to individuals with high gene count (Le Chatelier et al., 2013).

Some of the pathologies associated with high-fat diet associated with the western diet has been explained by the increased production of TMAO from gut microbiota derived TMA *via* the action of hepatic FMO3. Accumulation of TMAO is linked to atherogenesis, hyperactivation of platelets resulting in adverse CVD events such as stroke, heart attack and mortality (Tang et al., 2019). Although conclusive evidence is still required, there is evidence that supports that the Mediterranean diet lowers TMAO levels (Estruch et al., 2018) possibly through extra virgin oil’s 3,3-dimethyl-1-butanol (DMB) which lowers the production of TMAO (Tang et al., 2019). However, more studies are needed to establish the mechanistic link between TMAO levels, and its contribution to the pathogenesis of hypertension. There still remains significant variations among studies but the evidence for TMAO’s pathogenic contribution is strong (Coutinho-Wolino et al., 2021).

## Potential therapies in hypertension by alteration of gut microbiota

The study of the interplay between drugs and microbiota, termed pharmacomicrobiomics, has explored direct influence of microorganisms on drug response and *vice versa* (Elrakaiby et al., 2014). This also extends to the interactions of microbiota with antihypertensive drugs (Chen et al., 2022). As changes in gut microbiota are associated with hypertension, it is imperative that management of hypertension can also be directed towards alteration of gut microbiota and TMAO.

The reports that increasing TMAO has potential to increase blood pressure suggests that lowering blood TMAO concentrations can reduce BP levels and several studies have demonstrated this using probiotics, minocycline and enalapril (Khalesi et al., 2014; Yang et al., 2015; Janeiro et al., 2018). Antihypertensive drug interaction with gut microbiota has also been evidenced in experimental studies. Some studies have found that rats treated with angiotensin converting enzyme inhibitors (ACEIs) like enalapril showed a significantly lower plasma TMAO level and a trend towards higher 24-h urine excretion of TMA and TMAO (Konop et al., 2018). This finding signifies a potential protective role of ACEIs in CVD-risk and thus, may be considered in individuals with high TMAO levels or high TMAO producing bacteria. On the other hand, antihypertensive bioavailability can also be affected by gut microbiota (Yang et al., 2022b; Kyoung et al., 2022). For example, Yang et al. (2022b) found that *Coprococcus comes*, belonging to the genus Coprococcus (of the Firmicutes phylum), harboured esterase activity and degraded the ester ACEIs; quinapril and ramipril *in vitro*. In addition, Yang et al. (2022b) found that co-administration of quinapril with *C. comes* reduced the antihypertensive effect of quinapril in spontaneously hypertensive rats (SHR). On the other hand, Yoo et al. (2016) found that antibiotic intake increased the bioavailability of amlodipine by suppressing gut microbial metabolic activities, and likely affect the therapeutic efficacy. Yoo et al. (2016) found that incubation of amlodipine with human and rat feces increased the formation of amlodipine-associated metabolites and low levels of amlodipine, suggesting that gut microbiota may be key to the bioregulation of amlodipine. It is worth noting that understanding this role should guide in the management of hypertension. For example, antibiotics can increase the levels of certain drugs, and therefore this antihypertensive drug may need to be adjusted during concomitant treatment with antibiotics to avoid side effects.

Mounting evidence indicates that faecal microbiota transplantation also treats metabolic syndrome, diabetes, and hypertension (Li et al., 2017), and a potential therapy for treatment with supplementation in those with gut dysbiosis. Faecal microbiota transplantation from SHR to normal rats increased the systolic blood pressure of normal rats, and the converse was true (Toral et al., 2019). However, whether this can be used in humans is unknown. Moreover, faecal transplantation in humans raises a lot of ethical limitations.

Several human and animal studies have showed the long terms benefits of Lactobacilli species. For example, studies in mice revealed that administration of lactic acid bacteria such as Lactobacilli species has been demonstrated to reduce systolic blood pressure and vascular inflammation *via* increased NO production and reduction of NADPH oxidase-driven ROS production (Robles-Vera et al., 2018; Robles-Vera et al., 2020). While Lactobacilli species belongs to the Firmicutes phylum, these bacteria seem to reduce TMAO levels to prevent atherosclerosis and hypertension by unknown exact mechanisms (Zhao et al., 2021). However, as aforementioned, marine studies have established that the lactic acid produced by the bacteria is able to significantly reduce trimethylamine levels (Park et al., 2020), and the bacterial species are not included among those containing the choline trimethylamine-lyase (*CutC*) and its activating enzyme *CutD* gene responsible for TMAO production (Kalnins et al., 2015; Romano et al., 2015; Rath et al., 2017). A human study assessing the benefit of Lactobacili was a randomised, double blind, placebo-controlled study which showed that consumption of *Lactobacillus helveticus* fermented milk product reduced systolic blood pressure by 5.2 mmHg and diastolic blood pressure by 2.0 mmHg in 46 borderline hypertensive men (Mizushima et al., 2004).

On the other hand, other potential therapies targeting *CutC/D* gene cluster has been considered in the management of atherothrombosis (Roberts et al., 2018), which is associated with hypertension (Kumbhani et al., 2013). An animal model study by Roberts et al. (2018) showed that a single oral dose of a *CutC/D* inhibitor significantly reduced plasma TMAO levels for up to 3 days and rescued diet-induced enhanced platelet responsiveness and thrombus formation, without observable toxicity or increased bleeding risk. This was also supported by another study which showed that functional *CutC* levels in the intestinal microbiota can be transmitted by faecal transplantation, with a resultant high trimethylamine/TMAO generation thereby enhancing platelet reactivity and thrombosis potential within the recipient (Skye et al., 2018). *CutC/D* inhibition could be considered as a potential therapy to treat atherothrombotic heart disease, including hypertension.

Previous robust research on gut sodium absorption have provided some insights on therapeutic considerations in the treatment of hypertension and the role of gut microbiota. Sodium, due to the high solubility of its salts in the aqueous environment of the digestive tract, is almost completely absorbed into the enterocytes *via* sodium/glucose linked cotransporter (SGLT1, SLC5A1) and most importantly the sodium-hydrogen exchanger 3 (NHE3) (Curthoys, 2007). NHE3 is also found in the proximal renal tubule to regulate sodium and water absorption (Curthoys, 2007). Gut NHE3 plays the most important role in regulating sodium absorption, mainly in the small intestines, and when this sodium is filtered in the kidneys, more than 50% of it is reabsorbed by the renal NH3 in the proximal tubules (Nwia et al., 2022).

Studies evaluating the gut microbiota in NHE3-knock-out (NH3^−/−^) mice have shown a shift in the bacteria phyla with contraction of Firmicutes and expansion of Bacteroidetes along the gastrointestinal tract (terminal ileum, cecum, and distal colon) in NHE3^−/−^ compared to wild type (Engevik et al., 2013). This suggests that NHE3^−/−^ may play a role in reducing the Firmicutes/Bacteroidetes ratio, hence TMA and consequently TMAO production (Romano et al., 2015; Rath et al., 2017). Both animal and human studies have demonstrated increased sodium excretion when NHE3 inhibitors are used. Linz et al. (2012) performed an animal model study which showed that administration of an orally non-absorbable specific NHE3 inhibitor, SAR218034 (SAR), in SHR resulted in increased faecal content of sodium and water, decreased urinary sodium excretion and a reduction of the systolic blood pressure, suggesting the effect was mainly in the gastrointestinal tract. The blood pressure lowering effect was even additive when the NHE3 inhibitor was co-administered with an ACEI, ramipril (Linz et al., 2012). On the other hand, a similar effect was observed when the non-absorbable NH3 inhibitor, tenapanor, was used in both mice and humans (Spencer et al., 2014). These results were supported by another study which showed that global deletion of the NHE3 gene, including in the gastrointestinal system, significantly attenuated systolic blood pressure and mean arterial pressure responses to Ang II in both conscious and anesthetized NHE3^−/−^ mice (Li et al., 2015). Li et al. (2018) demonstrated that in both male and female mice, proximal tubule NHE3^−/−^ resulted in a significant promotion of the pressure natriuretic response and reduction in blood pressure compared to wild-type. Therefore, blood pressure lowering NHE3 inhibition could therefore be considered as a therapeutic target for hypertension, although more animal and human studies are needed to confirm that NHE3 inhibition is a treatment option, as some reports suggest that NHE3 deficiency can cause irritable bowel disease-like symptoms, gut dysbiosis, and an inflammatory immune system (Laubitz et al., 2016; Xue et al., 2022).

While the role of gut microbiota in human health has been a topic of interest for decades (Tannock, 2005; Sekirov et al., 2010), the intricacies of the role of the gut microbiota dependant trimethylamine N-oxide in the development and progression of hypertension has attracted interest and is a matter of ongoing research.

## Potential for plasma TMAO usage in clinical settings

Several studies have demonstrated the potential for TMAO to be used in clinical settings. For example, TMAO can be used as a prognostic biomarker in patients with pulmonary arterial hypertension where increased TMAO levels are associated with poor prognosis (Yang et al., 2022c). TMAO might be a useful biomarker in predicting CVD events (Guasti et al., 2021) such as plaque rupture in patients with ST-segment–elevation myocardial infarction (Tan et al., 2019) and all-cause mortality (Guasti et al., 2021). High levels of TMAO increases the risk of atherosclerotic cardiovascular disease (Lee et al., 2021) and also has potential to be used in predicting metabolic syndrome, adipose dysfunction and non-alcoholic fatty liver disease (Barrea et al., 2018), identifying CKD (Zixin et al., 2022), and identifying individuals at higher risk of prediabetes (Roy et al., 2020) and type 2 diabetes mellitus (Li et al., 2022).

If the associations around TMAO prove to be causal, then TMAO could be targeted to potentially reduce the risk associated with these conditions including the risk for hypertension and hypertension related adverse events.

## Conclusion

TMAO and gut microbiota play a critical role in propagating and sustaining cardiovascular risks such as hypertension through various mechanisms that are still not well understood. Future studies should concentrate efforts in considering these aspects to further drive the clinical management of CVDs and hypertension.

### What is known

• The gut microbiota contributes to CVD risk
• TMAO is associated with CVD risk and hypertension

### What is new

• There are several mechanisms by which plasma TMAO levels might influence the pathogenesis and progression of hypertension. Vascular injury and inflammation is the hallmark underlying this pathogenesis.
• Antihypertensive drugs can modulate blood pressure through interactions with the gut microbiota and TMAO
• Faecal microbiota transplantation is a promising intervention for future research
• High TMAO concentrations correlates with high BP levels
• Plasma levels of TMAO can be used as a predictive and prognostic biomarker for CVD, hypertension and metabolic diseases.
